# Characterization of Diarrheagenic Antimicrobial Resistant Escherichia coli Isolated From Pediatric Patients in Tehran, Iran

**DOI:** 10.5812/ircmj.12329

**Published:** 2014-04-05

**Authors:** Maryam Heidary, Hassan Momtaz, Mahboobeh Madani

**Affiliations:** 1 Department of Microbiology, Falavarjan Branch, Islamic Azad University, Falavarjan, IR Iran; 2 Department of Microbiology, Shahrekord Branch, Islamic Azad University, Shahrekord, IR Iran

**Keywords:** Escherichia coli, child, Multiplex Polymerase Chain Reaction

## Abstract

**Background:**

Acute infectious diarrhea is one of the most important causes of morbidity and mortality worldwide.

**Objectives:**

The objective of this study was to characterize antimicrobial resistant diarrheagenic Escherichia coli strains isolated from diarrheic children in Tehran, IR Iran.

**Patients and Methods:**

In total, 550 stool samples from diarrheic pediatric patients, aged less than 60 months, were collected and immediately transferred to the laboratory. Isolation and identification of E. coli strains was done using bacteriological methods. Antimicrobial susceptibility testing was performed using the disk diffusion technique. Multiplex PCR was used to detect aadA1, tetA, tetB, dfrA1, qnr, aac (3)-IV, sul1, blaSHV, CITM, cat1, and cmlA antibiotic resistance genes.

**Results:**

From the total of 550 fecal samples examined, 154 samples (28%) were positive for diarraheagenic E. coli. High rates of antibiotic resistance were seen against penicillin ﴾100%), ampicillin ﴾89.6%﴿ and tetracycline ﴾83.1%﴿. Resistance against ciprofloxacin was low ﴾28.6%﴿. The prevalence of different resistance genes in the studied strains varied from 96.10% for aadA1 gene to 40.25% for sul1 gene. The frequencies of aadA1, tetA, tetB, dfrA1, qnr, aac(3)-IV, sul1, blaSHV, CITM, cat1, and cmlA genes were 96.10%, 85.06%, 84.41%, 51.94%, 72.07%, 54.54%, 40.25%, 57.79%, 90.25%, 59.74% and 60.38%, respectively.

**Conclusions:**

Our results indicated that antibiotic resistance is increasing in diarraheagenic E. coli strains in Iran. It is imperative to develop strategies for prevention and control of resistant organisms. Changes in patterns of resistance against commonly used antibiotics in Iran indicate that an applied surveillance system and introduction of guidelines for appropriate antibiotic prescription are necessary.

## 1. Background

Acute infectious diarrhea (AID) is one of the most important causes of morbidity and mortality worldwide. This disease remains a major public health challenge, particularly in developing countries where it is a leading cause of death (1-3). A variety of microorganisms including bacteria, viruses and parasites can be associated with severe AID in children (4-7). Many studies have reported that among the bacterial pathogens, diearrheagenic Escherichia coli (E. coli) (DEC) is one of the most frequent causes of AID in children under five years old in developing countries (8-10). Previous studies have estimated that annually in Iran, diarrhea is responsible for 18 million cases of illness, 12 million medical visits, one million hospital admissions, and more than 500 deaths in children younger than five years old (10, 11). Also, according to the Global Burden Disease Report of the World Health Organization (WHO), diarrhea is the second most common cause of mortality in children under five years old (12-15).

Microbial resistance to antibiotics due to treatment of bacterial pathogens and extended use of different antibiotics is an increasing public health problem worldwide. Administration of antimicrobial agents has caused turbulence in the ecological balance between the host and microorganisms. In addition, acquisition of antibiotic resistance by pathogenic bacteria has increased and this may lead to more severe infections (7, 16-19). The intestinal E. coli microflora may provide an important reservoir for antibiotic-resistant bacteria and resistance genes. Presences of antimicrobial resistance genes have been reported previously for clinical E. coli isolates from humans and animals (20, 21). Genetic mutation and the role of genetic mobile elements such as plasmids, integrons and transposons are the most common ways for antibiotic resistance distribution (3, 8). Multidrug resistance to antimicrobial agents among bacterial pathogens is of great concern in clinical settings and is a major public health issue (21). Therefore, the ecological impact of different antimicrobial agents, as well as development of antimicrobial resistance before appearance in pathogenic strains and in clinical infections, could be studied in intestinal *E. coli*. Knowledge on antimicrobial resistance of diarrheagenic pathogens is important for selecting the appropriate therapy and necessary for physicians in order to treat infections using appropriate antibiotics (21, 22).

## 2. Objectives

The aim of this study was to characterize antimicrobial resistance in DEC strains isolated from pediatric patients in Tehran, IR Iran.

## 3. Patients and Methods

### 3.1. Clinical Specimens

This experimental study was carried out from March 2010 to September 2011. In total, 550 stool samples were collected randomly from children with diarrhea younger than 60 months old. The method of sample collection was simple accidental. These samples were collected from two major hospitals of Tehran, Iran (Baqyiatallah and Peyambar-Azam Hospitals). Information on some clinical and epidemiological features was obtained through questionnaires. These patients presented different types of symptoms including nausea, fever and dysentery. Stool specimens were collected using sterile swabs, and transported to the laboratory at 4°C using a cooler with iced-packs. Sample collection was performed with the consent of the children’s parent or guardian, and the study was approved by the local bioethical committee of Shahrekord Islamic Azad University. Control subjects included healthy children with no history of diarrhea for at least one month.

### 3.2. Isolation of Bacteria

Three milliliters of each sample was blended with 225 mL of nutrient broth (Merck, Germany) for two minutes at normal speed, using a Stomacher lab blender and incubated at 37°C for 24 hours. A 1 mL sample of the nutrient broth culture was mixed with 9 mL of MacConkey broth (Merck, Germany) and further incubated at 37°C for 24 hours. One loop of each tube was streaked on MacConkey agar (Merck, Germany). A typical purple colony of E. coli from each sample was streaked on a separate eosin methylene blue agar (EMB agar) plate (Merck, Germmany) and incubated at 37°C for 24 hours. A metallic green colony from each plate with a typical E. coli morphology was selected and examined by biochemical tests, including hydrogen sulfide, citrate, urease and indol based on the API test system (BioMerieux, Marcy L’Etoile, France) (23).

### 3.3. Antibiotic Susceptibility Testing

All E. coli isolates were tested for susceptibility to penicillin (10 u/disk); tetracycline (30 µg/disk); streptomycin (10 µg/disk); chloramphenicol (30 µg/disk); sulphonamides (100 µg/disk), sulfamethoxazol (25 µg/disk); gentamycin (10 µg/disk); lincomycin (2 µg/disk); cephalothin (30 µg/disk); trimethoprim (5 µg/disk); enrofloxacin (5 µg/disk); ciprofloxacin (5 µg/disk); ampicillin (10 u/disk); by the Kirby-Bauer disk diffusion method (MAST House, Merseyside, United Kingdom) (24). Resistance break points were those recommended by the Clinical and Laboratory Standards Institute (25), and was reported as sensitive or resistant based on break points. *Escherichia coli* ATCC 25922 was used as a quality control organism for antimicrobial susceptibility determination.

### 3.4. Detection of Antibiotic Resistance Genes

#### 3.4.1. DNA Extraction

DNA extraction was carried out using the boiling method. Briefly, the culture of each isolate was inoculated in Tryptose soy broth (Merck, Germany) and incubated at 37°C for 24 hours. Bacteria were harvested from an overnight broth culture and suspended in sterile water (DDW), after which the bacterial suspension was boiled for 10 minutes and chilled on ice and finally the debris were separated by centrifugation. The supernatant was taken as the DNA template for PCR.

#### 3.4.2. Primers and PCR Conditions

Multiplex PCR amplification was used to identify 11 antibiotic resistant genes including aadA1 for streptomycin (26); tetA, and tetB for tetracycline (26); dfrA1 for trimethoprim (27); qnr for fluoroquinolone (28); aac(3)-IV for gentamicin; sul1 for sulfonamide; blaSHV for cephalothin; CITM for ampicillin; and cat1 and cmlA for chloramphenicol resistance genes (29). Positive controls for the resistance genes were DNA samples from bacterial isolates previously characterized and sequenced in-house. The primers for all genes are presented in Table 1. Each PCR assay was carried out with a 50 µL mixture containing 10X PCR buffer, 2.5 mM MgCl2, 200 mM dNTP (Fermentas, Lithuania) 2.0 U of Taq DNA polymerase (Fermentas, Lithuania), 0.5 mM of each primer set and 3 µL of the DNA template. The specificity and optimal concentration of primers was determined experimentally. PCR was carried out on a thermocycler (Eppendrof Mastercycler 5330; Eppendorf-Nethel-Hinz GmbH, Hamburg, Germany) using the following cycle: after an initial denaturation cycle of seven minutes at 94ºC, the reaction mixes were subjected to 32 amplification cycles of 60 seconds at 95ºC and 70 second at 55ºC and two minutes at 72ºC, and final extension of five minutes at 72ºC. The reference strains served as the positive control and distilled water as the negative control. The PCR products were then separated by electrophoresis on a 2.0% agarose gel, stained with ethidium bromide and visualized using UV illumination. A 100 bp DNA molecular marker (Fermentas, Lithuania) was used to determine the size of the amplicons.

**Table 1.** Primers Used for Detection of Common Antibiotic Resistance Genes of Diarrheagenic *E. coli*

Primer	Sequence	Amplification Size	Antibiotic Resistance	Reference
**aadA1**				
F	TATCCAGCTAAGCGCGAACT	447	streptomycin resistance	(26)
R	ATTTGCCGACTACCTTGGTC			
**tetA**				
F	GGTTCACTCGAACGACGTCA	577	tetracycline resistance	(26)
R	CTGTCCGACAAGTTGCATGA			
**tetB**				
F	CCTCAGCTTCTCAACGCGTG	634	tetracycline resistance	(26)
R	GCACCTTGCTGATGACTCTT			
**dfrA1**				
F	GGAGTGCCAAAGGTGAACAGC	367	trimethoprim resistance	(27)
R	GAGGCGAAGTCTTGGGTAAAAAC			
**Qnr**				
F	GGGTATGGATATTATTGATAAAG	670	fluoroquinolone	(28)
R	CTAATCCGGCAGCACTATTTA		resistance	
**aac(3)-IV**				
F	CTTCAGGATGGCAAGTTGGT	286	gentamicin resistance	(29)
R	(TCATCTCGTTCTCCGCTCAT			
**Sul1**				
F	TTCGGCATTCTGAATCTCAC	822	sulfonamide resistance	(29)
R	ATGATCTAACCCTCGGTCTC			
**blaSHV**				
F	TCGCCTGTGTATTATCTCCC	768	cephalothin resistance	(29)
R	CGCAGATAAATCACCACAATG			
**CITM**				
F	TGGCCAGAACTGACAGGCAAA	462	ampicillin resistance	(29)
R	TTTCTCCTGAACGTGGCTGGC			
**ereA**				
F	GCCGGTGCTCATGAACTTGAG	419	erythromycin	(29)
R	CGACTCTATTCGATCAGAGGC		resistance	
**cat1**				
F	AGTTGCTCAATGTACCTATAACC	547	chloramphenicol	(29)
R	TTGTAATTCATTAAGCATTCTGCC		resistance	
**cmlA**				
F	CCGCCACGGTGTTGTTGTTATC	698	chloramphenicol	(29)
R	CACCTTGCCTGCCCATCATTAG		resistance	

### 3.5. Statistical Analysis

The data were analyzed using the SPSS (statistical package for the social sciences) software and P values were calculated using Chi-square and Fisher's exact tests to determine significant relationships between various criteria and distribution of antibiotic resistance properties of STEC strains isolated from diarrheic pediatric patients. A P value less than 0.05 was considered statistically significant.

### 3.6. Ethical Issues

The present study was accepted by the ethical committee of the Baqyiatallah and Peyambar-Azam Hospitals of Tehran, Iran and Microbiology and Infectious Diseases Center of the Islamic Azad University of Shahrekord, Iran. Written informed consent was obtained from all of the studied patients or their parents. The agreement between the Islamic Azad University of Shahrekord and the two mentioned hospitals had been signed before sampling (on 10th of April and 15th of April 2013).

## 4. Results

A total of 550 rectal swabs were examined for the presence of diarrheagenic *E. coli* strains isolated from children between the ages of one month to five years. Totally, 154 samples (28%) were positive for diarrheagenic *E. coli*. The greatest rates for isolates recovery were from the 13-24 month age group (38.3%), followed by the 1-12 month age group (23.38%) (Table 2). Incidence of nausea, fever and dysentery clinical signs were 69 (44.8%), 64 (41.6%) and 21 (13.6%), respectively. From a total of 154 positive diarrheagenic *E. col*i strains, 74 (48 %) and 80 (51.9%) were isolated from female and male patients, respectively (Table 2). In total, 47 (30.5%), 62 (40.2%), and 45 (29.2%) positive specimens were isolated from the samples, which were collected during spring, summer and fall, respectively. Antibiotic susceptibility testing showed high antibiotic resistance rates against penicillin (1002%), ampicillin (89.6%), tetracycline (83.1%), trimethoprim (71.4%) and streptomycin (50.6%) antibiotics. Antibiotic resistance rates against enrofloxacin (33.1%) and ciprofloxacin ﴾28.6%) were the minimum. Table 3 shows the complete results of antimicrobial resistance among diarrheagenic *E. coli*.

**Table 2.** Description of Different Criteria of Patients in Order of Gender (n = 154) ^a^

Criteria (Age, Season and Clinical Signs)	Male	Female	Results
**1-12**	20	16	36 (23.4)
**13-24**	31	28	59 (38.3)
**25-36**	11	13	24 (15.6)
**37-48**	6	10	16 (10.4)
**49-60**	12	7	19 (11.7)
**Spring**	23	24	47 (30.5%)
**Summer**	30	32	62 (40.2%)
**Fall**	24	21	45(29.2%)
**Nausea**	36	33	69 (44.8%)
**Fever**	36	28	64(41.6%)
**Dysentery**	12	9	21(13.6%)

^a^ Data are presented as No. (%).

**Table 3.** Antibiotic Resistance Profile of Diarrheagenic E. coli in Iran (Disk Diffusion Method) (n = 154)

*E. coli*	P10	TE30	S10	C30	SXT	GM10	E15	NFX5	L2	CF30	CIP5	TMP5	AM10	SF100
**Total**	142	128	78	92	60	79	111	51	50	88	44	62	138	83

All of the isolates had resistance to more than one antibiotic. There was a significant statistical difference for incidence of bacteria between 13-24 month old and 37-48 month old pediatrics (P < 0.05). Also, differences were significant (P < 0.05) for bacterial resistance between ampicillin and penicillin with ciprofloxacin and enrofloxacin (P < 0.05). However, there were no significant statistical differences between the sex of the patients and rates of antibiotic resistance. There were no significant differences for incidence of bacteria between the male and females. The results obtained from analysis of the antimicrobial resistance genes showed that 11 resistance genes were identified in all E. coli strains. The distribution of resistance genes among E. coli isolates is summarized in Table 4. The prevalence of different resistance genes varied from 96.10% for the aadA1 gene to 40.25% for the sul1 gene. Trimethoprim resistance attributed to dfrA1 gene was found in 51.94% of the strains. Of the tetracycline resistant isolates, 85.06% were positive for tetA genes while 84.41% were positive for tetB. The catI and cmlA genes were detected in 59.74% and 60.38% of chloramphenicol resistant isolates, respectively. The SHV β-lactamase gene was identified in 57.79% (89/154) of cephalithin resistant isolates. For ampicillin resistance, 90.25% of the isolates were positive for the CITM gene. The sul1 gene was detected in 40.25% of sulfonamide resistant isolates. The aac(3)-IV gene which codes resistance against gentamycin was found in 54.54% of isolates (Table 4).

**Table 4.** Distribution of Antimicrobial Resistance Genes in Diarrheagenic E. coli in Iran (n = 154)

Total Positive Samples	Antibiotic Resistance Genes, %										
	aadA1	tetA	tetB	dfrA1	qnr	aac(3)-IV	sul1	blaSHV	CITM	cat1	cmlA
***E. coli ***	148	131	130	80	111	84	62	89	139	92	93
	(96.10)	(85.06)	(84.41)	(51.94)	(72.07)	(54.54)	(40.25)	(57.79)	(90.25)	(59.74)	(60.38)

## 5. Discussion

DEC has been identified as one of commensally pathogenic bacteria, which cause diarrhea among infants and children, especially in developing countries (4, 30, 31). Many investigations have been previously done in order to survey the prevalence of DEC in different countries. Albert et al., (32) found that the frequency of DEC in Bangladeshi children was higher than 40%. In another study, Shehabi et al. (33) reported that the prevalence of DEC isolates was 34% among children with diarrhea in Jordan. In the present study, of the 550 stool samples examined, DEC strains were isolated from 28% of cases. This finding is interesting however a previous study in Tehran reported the prevalence rate of DEC as 54% (3). The possessive increase in antimicrobial resistance of bacterial strains is becoming of concern, particularly in developing countries (34). The observed antimicrobial resistance may be a result of overuse and misuse of different antibiotics in infection treatments (1, 25). Commensal bacteria are the main source of resistance genes for pathogenic bacteria (34). The DEC is one of these commensal bacteria, and *E. coli* have been often used as useful indicators for survey of the selective pressure of antimicrobial agents and distribution of acquired resistance genes (6, 7, 10, 12, 14, 25). Previous studies have indicated that the majority of DEC strains have shown antibiotic resistance at least against one of the following antibiotics sulfonamide, cotrimoxazole, and ampicillin (18). In developing countries, some antibiotics are widely used to treat infectious diseases because of their low cost and availability (14, 19). Currently, trimethoprim-sulfamethoxazole, ampicillin and tetracycline are frequently used to treat diarrhea (7).

In our study, a high frequency of DEC antimicrobial resistance to commonly used antibiotics such as ampicillin (89.6%) was observed. This finding is comparable to previously reported studies on children from Peru and Bolivia, Vietnam, Tanzania, Mexico, Argentina and Mozambique that reported a prevalence of ampicillin resistance of more than 70% in DEC isolates (2). In another study (7) in Honoi, Vietnam, DEC strains exhibited different levels of resistance to ampicillin (86.4%), chloramphenicol (77.2%) and trimethoprim-sulfamethoxazole (19.1%). In another study, antibiotic resistance rates among Egyptian DEC isolates were 68.2%, 57.2% and 24.2% for ampicillin, trimethoprim-sulfamethoxazole and ampicillin-sulbactam, respectively (35). Bouzari et al. (19) found high resistance rates against sulfamethoxazole-trimethoprim, tetracycline and chloramphenicol in DEC strains isolated from Tehran, IR Iran. With the exception of sulfamethoxazole, our findings are in agreement with the report of Bouzari et al. (19). Many studies indicate that multidrug resistant E. coli are widespread among the DEC strains and occurrence of resistant DEC could be because of environmental conditions, including transmission of resistant isolates from adults to children, or from animals to humans (3). Multidrug resistant DEC strains have been reported in previous studies (8, 11, 12). EI Metwally et al. (36) reported that 56% of DEC isolates were multidrug resistant (simultaneous resistance against SXT, ampicillin and tetracycline). Also, Souza et al. (37) found a high rate of antimicrobial drug-resistance (65%) among DEC strains isolated from children. In our study, all of the isolates were multidrug resistant. Our study revealed a high prevalence of multidrug antibiotic resistance among E. coli isolates, because of overuse and misuse of antibiotics to treat diarrheal diseases in Iran. The main factors for dissemination of resistance genes are mobilizable plasmids, self-transmissible plasmids and conjugative transposons (38).

In this present study, among tetracycline resistance strains, 85.06% and 84.41% harbored tetA and tetB gene, respectively. Gene carriage for ampicillin resistance gene (CITM) was observed in 90.25% of ampicillin resistant isolates. However, this speculation does not apply to streptomycin, which has had limited clinical use for many years; nevertheless, the high rate of resistance (78.2%) to this drug has persisted (38). Possible explanation for the persistence of resistance to these drugs includes the properties of the mobile elements on which the determinants are carried, and the potential selection pressures other than antibiotics for human medical use (3). According to the result of this study, resistance to ampicillin, tetracycline, trimethoprim, and chloramphenicol were very common in DEC. It seems that the patients infected with *E. coli* strains, could be a potential source for antibiotics resistance genes (34).

In the recent years, molecular methods have been reported as rapid and sensitive techniques. Multiplex PCR is a rapid and specific diagnostic method for simultaneous detection of several virulence and antimicrobial resistance genes in one PCR reaction (39-41). Studies have been carried out in order to study the prevalence of antibiotic resistance genes in E. coli isolates of animal and human samples. Momtaz et al. (42) detected tetA, tetB, dfrA1, qnrA, catA1, cmlA, sul1 and ereA genes in *E. coli* isolated from slaughtered commercial chickens by multiplex PCR. They reported the distribution of studied genes as: tetA and tetB ﴾52.63%﴿, dfrA1, qnrA, catA1 and cmlA ﴾36.84%) and sul1 and ereA ﴾47.36%). Ahmed et al. (43) studied the frequency of antimicrobial resistance genes in equine fecal E. coli strains by PCR in North West England. They found high rates of dfr, tem beta-lactamase, tet and cat genes (93%, 91%, 89% and 73.5%, respectively). In the present study, the occurrence of 11 antibiotic resistance genes in DEC strains was investigated by multiplex PCR. The frequency of aadA1, tetA, tetB, dfrA1, qnr, aac(3)-IV, sul1, blaSHV, CITM, cat1, and cmlA genes were in 96.10%, 85.06%, 84.41%, 51.94%, 72.07%, 54.54%, 40.25%, 57.79%, 90.25%, 59.74%, and 60.38%, respectively.

According to the progressive increase in antibiotic resistance among enteric pathogens, particularly in developing countries, it is imperative to implement strategies to prevent and control the emergence and spread of resistant organisms by inexpensive, sensitive, and simple methods. Practitioners should pay more attention to antibiotic prescription especially that related to diarrhea in children.
